# Association of In-Hospital Mortality and Dysglycemia in Septic Patients

**DOI:** 10.1371/journal.pone.0170408

**Published:** 2017-01-20

**Authors:** Hsiao-Yun Chao, Peng-Hui Liu, Shen-Che Lin, Chun-Kuei Chen, Jih-Chang Chen, Yi-Lin Chan, Chin-Chieh Wu, Gerald N. Blaney, Zhen-Ying Liu, Cho-Ju Wu, Kuan-Fu Chen

**Affiliations:** 1 Department of Emergency Medicine, Chang Gung Memorial Hospital, Linkou, Taiwan; 2 Department of Emergency Medicine, Chang Gung Memorial Hospital, Keelung, Taiwan; 3 Clinical Informatics and Medical Statistics Research Center, Chang Gung University, Taoyuan, Taiwan; 4 Community Medicine Research Center, Chang Gung Memorial Hospital, Keelung, Taiwan; Azienda Ospedaliero Universitaria Careggi, ITALY

## Abstract

**Background:**

The associations between dysglycemia and mortality in septic patients with and without diabetes are yet to be confirmed. Our aim was to analyze the association of diabetes and sepsis mortality, and to examine how dysglycemia (hyperglycemia, hypoglycemia and glucose variability) affects in-hospital mortality of patients with suspected sepsis in emergency department (ED) and intensive care units.

**Methods:**

Clinically suspected septic patients admitted to ED were included, and stratified into subgroups according to in-hospital mortality and the presence of diabetes. We analyzed patients’ demographics, comorbidities, clinical and laboratory parameters, admission glucose levels and severity of sepsis. Odds ratio of mortality was assessed after adjusting for possible confounders. The correlations of admission glucose and CoV (blood glucose coefficients of variation) and mortality in diabetes and non-diabetes were also tested.

**Results:**

Diabetes was present in 58.3% of the patients. Diabetic patients were older, more likely to have end-stage renal disease and undergoing hemodialysis, but had fewer malignancies, less sepsis severity (lower Mortality in Emergency Department Sepsis Score), less steroid usage in emergency department, and lower in-hospital mortality rate (aOR:0.83, 95% CI 0.65–0.99, p = 0.044). Hyperglycemia at admission (glucose≥200 mg/dL) was associated with higher risks of in-hospital mortality among the non-diabetes patients (OR:1.83 vs. diabetes, 95% CI 1.20–2.80, p = 0.005) with the same elevated glucose levels at admission. In addition, CoV>30% resulted in higher risk of death as well (aOR:1.88 vs. CoV between 10 and 30, 95%CI 1.24–2.86 p = 0.003).

**Conclusions:**

This study indicates that while diabetes mellitus seems to be a protective factor in sepsis patients, hyper- or hypoglycemia status on admission, and increased blood glucose variation during hospital stays, were independently associated with increased odds ratio of mortality.

## Introduction

The global incidence of severe sepsis increased substantially over the last two decades [1, 2]. Depending on the time point and the outcome measured, severe sepsis and septic shock killed one-fourth to one-half of hospitalized septic patients. In Taiwan, the incidence of severe sepsis was 3.90 per 1,000 person-years [3]. Among the 683,421 severe sepsis patients identified from the nationwide population-based cohort from year 2000 to 2010, 229,792 (33.6%) patients died in Intensive Care Units (ICU) during hospitalization for sepsis [4]. Further analysis of hospital-based data in Taiwan shown that hospitalizations with severe sepsis in ICUs were associated with substantial 28-day mortality, as high as 61% [5], despite with all the advances in pharmacotherapy and increasing adoption of bundle resuscitation program to optimize the supportive care quality over time.

Diabetes is an increasingly common illness. In a Taiwanese population-based study, 27.7% of the severe septic patients admitted to ICUs were comorbid with diabetes [6]. Generally, patients with diabetes have an increased risk of developing common infections compared with non-diabetic patients [7, 8]. It is thus logical to hypothesize that this comorbidity of diabetes may also precipitate the development of severe sepsis or septic shock, as both diabetes and sepsis are associated with systemic or excessive vascular endothelial activation; which may result in organ hypoperfusion or dysfunction, a potential risk factor for sepsis mortality [9]. However, some recent studies have shown that diabetes might not be associated with increased mortality in patients with sepsis [6, 10]. Furthermore, no harmful association between diabetes and mortality can be found in patients across different sepsis severities. Another Taiwanese cohort, derived from the national health insurance database [6] between 1998 and 2008 with septic patients admitted to ICUs, also drew similar conclusions that patients with diabetes did not demonstrate a worse outcome than those who had no diabetes history.

On the other hand, acute hyperglycemia at admission was associated with increased hospital mortality in critically ill patients [11], with non-diabetic patients more vulnerable to high initial glucose level at emergency department (ED) admission in a non- critically ill patient cohort [12]. Also, many large studies demonstrate that increasing glycemic variability conferred a strong independent risk of mortality in critically-ill patients [13–15].

Because different study cohorts, population definitions, measured time points, or analytic methods may give discrepant results; whether the presence of diabetes, the dysregulated metabolic homeostasis as reflect in admission hyperglycemia, hypoglycemia, or excessive glucose variation during ED admission, or any or all of these dysglycemia constitute independent risks in mortality for patients with sepsis, remains to be clarified. The purpose of this study is to identify if diabetes, or having admission hyper- or hypoglycemia and excess blood glucose fluctuation will alter the risk of in-hospital mortality for patients with clinically suspected sepsis.

## Materials and Methods

### Study design and setting

Patients were retrospectively identified from the electronic medical record of approximately 180,000 ED visits at a tertiary medical center in 2010, after the study was approved by the Institutional Review Board of the Chang Gung Memorial Hospital, which waived the requirement of informed consent.

### Study population and definition

Patients with clinically suspected sepsis were eligible based on the availability of at least two blood culture ordered by emergency physicians. Diabetes was defined as those having an ICD-9-CM (International Classification of Disease, 9^th^ Revision Clinical Modification) code of “250.XX” in their medical records, appearing at least twice in their outpatient clinics visits or ED records, and/or at least once in the inpatient records, within the previous six months. To verify the representativeness of the diabetes cohort, the prescription claims database was linked to check if these patients were already receiving anti-diabetic treatments. The subgroup for glucose variation evaluation was formed by extracting from the total cohort those with at least two blood glucose tests within 48 hours after ED admission. The coefficients variation (CoV) of blood glucose was defined as the ratio of the standard deviation to the mean of blood glucose values, obtained within 48 hours after admission. Additional sepsis-3 subgroups were constructed by using the recently updated sepsis-3 definition, which re-defines sepsis as evidence of infection plus acute increase of Sequential Organ Failure Assessment (SOFA) Score (delta SOFA) ≥2 [16]. The severity of sepsis was shown based on the presence of Systemic Inflammatory Response Syndrome (SIRS, defined as two or more criteria of increased heart rate or respiratory rate, and elevated or depressed body temperature or white blood cell count), the MEDS (Mortality in Emergency Department Sepsis) score [17], the presence of bacteremia, or ICU admission. The primary outcome was in-hospital mortality.

### Data acquisition

Electronic medical records have been implemented in our institution since 2004, including medical history, clinical and laboratory findings, diagnostic images, prescription and management. Prior to data collection, variables were defined and converted to standard formats. To extract large amounts of data from the database, structured query language (SQL) was employed to efficiently retrieve the medical records of all the eligible cases. This programming language enables the managing of data streams held in a relational database, which were then stored in Microsoft Access (Microsoft; Redmond, WA, USA). All the queries obtained were subsequently examined by manual chart review and discrepancies were resolved separately by two emergency department physicians. Basic demographics, underlying illnesses, laboratory findings, Systemic Inflammatory Response Syndrome (SIRS), MEDS (Mortality in Emergency Department Sepsis) score, severity of sepsis (i.e. bacteremia, ICU admission, and sepsis-3), steroid rescue use in ED and discharge status were collected.

### Statistical analysis

The data are presented as median and interquartile range (IQR), or number and percentage. All analyses were performed with Stata statistical software (version 13.1; Stata Corp, College Station, TX, USA). A two-sided p<0.05 was regarded as statistically significant. The Chi-square test was used for binary variables and Wilcoxon Rank-sum test was used for continuous variables for subgroups comparisons. Multivariable logistic regression was performed to test the association between in-hospital mortality and admission blood glucose, and the presence of diabetes adjusting other comorbidities. In addition, to test how well the regression models fit the data, the Hosmer-Lemeshow Goodness-of-fit tests were applied. We also utilize the area under the receiver operating characteristics curve (AUC) to evaluate the discriminative capacity of the model. Glucose variation (Coefficient of Variation, CoV), with cut-off thresholds set at <10%, and >30%, and their associations with in-hospital mortality after adjusting the age, admission blood glucose, the presence of diabetes and diagnosis of sepsis-3 were analyzed with multivariate logistic regression. Interaction between glucose CoV and admission glucose was tested. The p value and the 95% confidence interval (95% CI) for the adjusted odds ratio (aOR) were calculated.

### Subgroup analysis

Since stronger correlation with mortality had recently been established by using sepsis-3 rather than the previous definition of SIRS, our clinically suspected sepsis patients, in addition to defined by two sets of blood culture plus a confirmed focus of infection, were further stratified into subgroups according to the presence of sepsis-3, to examine whether discrepancies in mortality outcomes exist among different subgroups, following multivariate logistic regression.

## Results

**Fig 1:**
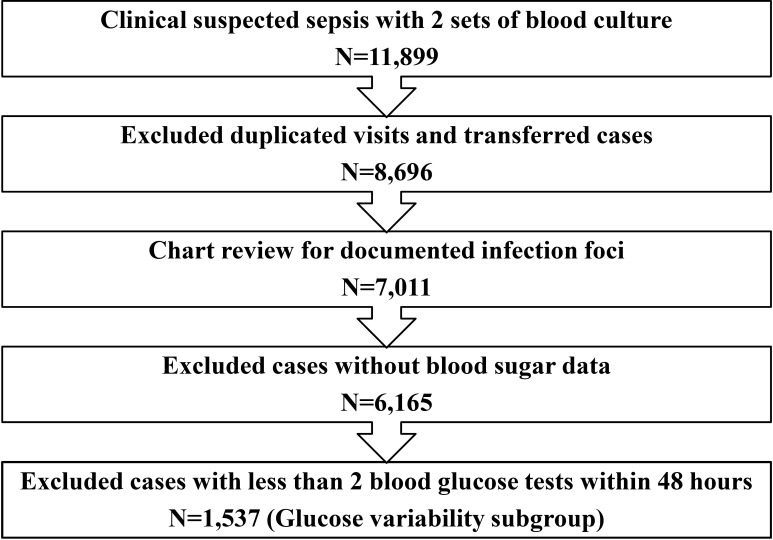
Patient flow chart.

The patient flow is depicted in Fig 1. From the preliminary cohort of 11,899 patients with clinically suspected sepsis who had at least two sets of blood culture tests, excluding the duplicated visits and referred cases, and only including patients who visit the ED for the first time during the study period, resulted in 7,011 cases with documented infection focus. Further exclusion of patients with unavailable blood glucose data resulted in a 6,165-patient cohort. In addition, a subgroup of 1,537 patients for the analysis of glucose variability was constructed by excluding those with less than two blood glucose tests during the 48 hours after ED admission (S1 Table).

The demographics, comorbidities, laboratory parameters, and severity of sepsis of all study patients and subgroups are shown in Table 1. The median age was 66 years (IQR: 51–78); 58.4% had diabetes, and 77.4% of the diabetic patients were prescribed with either oral anti-diabetic agents, insulin or combination therapy. Among all the patients, 7.1% died during the hospital stays, those admitted to ICU had a mortality rate as high as 27.3%. Compared with the survivors, the non-survivors were older, more likely to have underlying illnesses and malignancies, and had greater abnormalities in laboratory parameters but less likely to be diabetic patients. Compared with the non-diabetic patients, the diabetic patients were older, more likely to be female, more likely to have end-stage renal disease and undergoing hemodialysis, but less likely to have malignancy (17.7% vs. 26.6%), to develop sepsis-3 (delta SOFA≥2, 45.5% vs. 47.3%) and to have ICU admission (6.3% vs. 7.5%). Other sepsis related severity indices, including SIRS criteria, MEDS score and presence of bacteremia, all favored the diabetic group.

**Table 1:** Demographic, comorbidities, laboratory, sepsis severity of the study patients, stratified by survivor vs. non-survivor; diabetes vs. non-diabetes.

	All patients (N = 6165)		Non-survivor(N = 437)		Survivor (N = 5728)					Diabetes (N = 3594)		Non-diabetes (N = 2562)				
	Median or N	(IQR) or %	Median or N	(IQR) or %	Median or N	(IQR) or %	p-value	OR*	95%CI	Median or N	(IQR) or %	Median or N	(IQR) or %	p-value	OR**	95%CI
**Demographic & comorbidities**																
Age	66	(51–78)	72	(56–81)	66	(50–78)	<0.001	1.020	(1.01–1.03)	66.5	(52–78)	65	(48–79)	<0.001	1.004	(1.001–1.007)
>65 years	3134	50.8	268	61.3	2866	50	<0.001	1.580	(1.30–1.93)	1865	51.9	1269	49.4	<0.05	1.110	(1.000–1.225)
Male	3337	54.1	254	58.1	3083	53.8	<0.05	1.190	(0.98–1.45)	1877	52.2	1460	56.8	<0.01	0.830	(0.750–0.920)
Diabetes	3594	58.3	225	51.5	3369	58.8	<0.01	0.740	(0.61–0.90)	3594	100	0.0	0.0	<0.001		
Chronic kidney diseases	772	12.5	69	15.8	703	12.3	<0.05	1.340	(1.02–1.75)	540	15.0	232	9.0	<0.001	1.780	(1.520–2.100)
Hemodialysis	432	7	43	9.8	389	6.8	<0.05	1.500	(1.08–2.09)	285	7.9	147	5.7	<0.01	1.420	(1.160–1.740)
Malignancy	1351	21.9	213	48.7	1138	19.9	<0.001	3.840	(3.14–4.68)	635	17.7	685	26.6	<0.001	0.590	(0.520–0.670)
Chemotherapy	525	8.5	83	19	442	7.7	<0.001	2.800	(2.17–3.63)	225	6.3	296	11.5	<0.001	0.510	(0.430–0.620)
**Laboratory**																
Platelet, 10^3^/uL	204	(148–270)	170	(96–271)	205	(151–270)	<0.001	0.998	(0.99–0.99)	206	(154–273)	200	(139–263)	<0.001	1.001	(1.000–1.001)
WBC, 10^3^/uL	11.2	(7.8–15.1)	12.1	(7.7–16.5)	11.2	(7.8–15)	<0.05	1.010	(1.00–1.02)	11.5	(8.2–15.3)	10.7	(7.3–14.9)	<0.05	1.004	(0.998–1.011)
**Sepsis severity**																
MEDS	6	(3–9)	9	(6–11)	6	(3–9)	<0.001	1.170	(1.14–1.21)	6	(3–9)	6	(3–9)	<0.001	0.960	(0.950–0.980)
SIRS>2	4312	69.9	332	76	3980	69.5	<0.01	1.390	(1.11–1.74)	2419	67.3	1893	73.6	<0.001	0.740	(0.660–0.820)
Fever	5793	94	394	90.2	5399	94.3	<0.01	0.560	(0.40–0.78)	3376	93.9	2417	94.0	<0.05	0.990	(0.800–1.220)
Bacteremia	1244	20.2	129	29.5	1115	19.5	<0.001	1.730	(1.39–2.15)	693	19.3	551	21.4	<0.05	0.880	(0.770–0.990)
Sepsis-3	2853	46.3	324	74.1	2529	44.2	<0.001	3.630	(2.91–4.52)	1636	45.5	1217	47.3	<0.05	0.930	(0.840–1.030)
Steroid usage in ER	573	9.3	53	12.1	520	9.1	<0.05	1.440	(1.02–2.04)	288	8.0	285	11.1	<0.001	0.700	(0.590–0.830)
Steroid usage in OPD	66	1.1	2	0.5	64	1.1	<0.05	0.690	(0.17–2.86)	32	0.9	34	1.3	<0.05	0.670	(0.410–1.090)
ICU admission	421	6.8	115	27.3	306	5.3	<0.001	6.330	(4.97–8.06)	228	6.3	193	7.5	<0.05	0.830	(0.680–1.020)

IQR, Interquartile Range; WBC, White Blood Cell Count; MEDS, Mortality in Emergency Department Sepsis Score; SIRS, Systemic Inflammatory Response Syndrome; Sepsis-3, evidence of infection plusΔSOFA (Sequential Organ Failure Assessment) score≥2

* Odds ratio of non-survivor vs. survivor

** Odds ratio of diabetes vs. non-diabetes.

Table 2 depicts the association between admission glucose levels and mortality. Those with glucose≤100 mg/dL demonstrated higher mortality rate than those with glucose ≥ 150 mg/dL at admission (11.0% vs. 7.7%, p = 0.005). After stratifying the patients into subgroups according to admission glucose level, an increasing trend of mortality rates was found in non-diabetic patients but not in the diabetic group (≥200 mg/dL: 15% vs. 7%, p = 0.005; ≥250 mg/dL: 20% vs. 7%, p = 0.005 and ≥ 300 mg/dL: 28% vs. 8%, p = 0.008). To adjust for the between group sepsis severity, we excluded patients not meeting sepsis-3 criteria, and stratified the study cohort into diabetes with sepsis-3 and non-diabetes with sepsis-3 into subgroups, to repeatedly verify the association between diabetes and non-diabetes with mortality (Table 3). Increased mortality and similar trends of risk were found between the two groups (≥200 mg/dL: 18.1% vs. 9.8%, p = 0.004; ≥250 mg/ dL: 20.3% vs. 11%, p = 0.001, and ≥ 300 mg/ dL: 32.3% vs. 11.8% p = 0.008).

**Table 2:** Associations between admission glucose levels and mortality in diabetes vs. non-diabetes patients.

	Diabetes (n = 3,594)		Non-diabetes (n = 2,571)				
	N (%)	Non-survivor	N (%)	Non-survivor	OR*	95%CI	p-value
**Admission glucose≤100 mg/dL**	386(10.7)	41(11.0)	360(14.0)	41(11.0)	0.71	(0.46–1.10)	0.130
**Admission glucose≥150 mg/dL**	1,756(48.9)	114(6.0)	651(25.3)	71(11.0)	1.15	(0.85–1.56)	0.350
**Admission glucose≥200 mg/dL**	1,111(30.9)	76(7.0)	205(8.0)	30(15.0)	1.83	(1.20–2.80)	0.005
**Admission glucose≥250 mg/dL**	756(21.0)	56(7.0)	97(3.8)	19(20.0)	2.13	(1.26–3.59)	0.005
**Admission glucose≥300 mg/dL**	527(14.7)	42(8.0)	47(1.8)	13(28.0)	2.33	(1.25–4.34)	0.008

CI, confidence interval

* Odds ratio of death of non-diabetes vs. diabetes.

**Table 3:** Associations between admission glucose levels and mortality in diabetes with sepsis-3 vs. non-diabetes with sepsis-3 patients.

	Diabetes with sepsis-3 (n = 1,636)		Non-diabetes with sepsis-3 (n = 1,217)				
	N (%)	Non-survivor	N (%)	Non-survivor	OR*	95%CI	p-value
**Admission glucose≤100 mg/dL**	172(10.5)	36(20.9)	166(13.6)	29(17.5)	0.92	(0.56–1.51)	0.750
**Admission glucose≥150 mg/dL**	957(58.5)	94(9.8)	379(31.1)	54(14.2)	1.31	(0.93–1.85)	0.120
**Admission glucose≥200 mg/dL**	633(38.7)	62(9.8)	127(10.4)	23(18.1)	2.04	(1.25–3.32)	0.004
**Admission glucose≥250 mg/dL**	429(26.2)	47(11.0)	64(5.3)	13(20.3)	2.74	(1.48–5.09)	0.001
**Admission glucose≥300 mg/dL**	289(17.7)	34(11.8)	31(2.5)	10(32.3)	2.56	(1.26–5.21)	0.008

Sepsis-3, evidence of infection plusΔSOFA (Sequential Organ Failure Assessment) score≥2; CI, confidence interval

* Odds ratio of death of non-diabetes with sepsis-3 vs. diabetes with sepsis-3.

**Fig 2:**
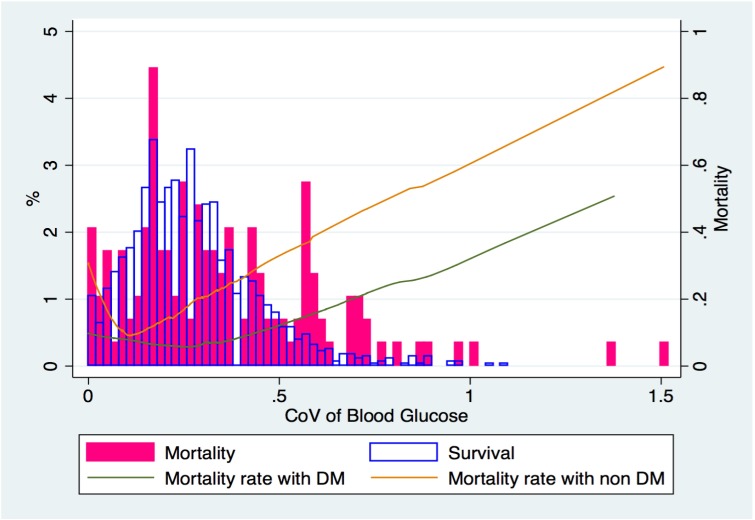
Distribution of in-hospital mortality according to blood glucose CoV in diabetes and non-diabetes.

Associations of glucose variation between diabetes, non-diabetes, and in-hospital mortality were demonstrated in Fig 2. The U-shaped association between CoV and mortality indicated septic patients with either low (<10%) or high CoV (>30%) had worse outcomes than those with CoV between 10% and 30% (11% or 12% vs. 7%, respectively). The non-diabetes patients seemed to be less tolerant to high glucose variation than those with diabetes.

By using multivariable logistic regression model, age≥65, malignancy, hemodialysis, and liver diseases were found to be associated with increased risk of mortality. Additionally, admission blood glucose ≤100 mg/dL or ≥200 mg/dL were associated with 2.04 and 1.54 times elevated aOR (adjusted Odds Ratio) of mortality, respectively. Infection sites of genitourinary, skin and abdominal origins have reduced risks of mortality than other infections (Tables 4 and 5). Among the glucose variability subgroup, most patients were diabetic (83.3% of this cohort were diabetes, see S1 Table), the impact of abnormal admission glucose on mortality was not significant (p = 0.611 & 0.090 for ≥200 mg/dL & ≤100 mg/dL, respectively, Table 5). However, patients with glucose CoV >30% (88.3% were diabetes) had a 1.88-time higher risk of death (p = 0.003), compared to those with glucose variation between 10% and 30%. Those with CoV<10% (75.4% were diabetes) also had numerical elevated risk of mortality (aOR = 1.36), although the p-value was not significant due to the small sample size of this subgroup (Table 5), and no interaction between CoV and abnormal admission glucose (≤100 mg/dL or ≥200 mg/dL) was found in the multivariate model. On the contrary, the presence of diabetes was associated with a 17% reduced risk of mortality (aOR: 0.83, *p* = 0.044) from the total patient cohort (table 4) and even greater reduced risk of mortality (43% reduction, aOR: 0.57, p = 0.009) from the glucose variability cohort (Table 5) indicating diabetic patients had better prognosis than non-diabetic patients. The Goodness-of-fit tests indicated that the models were correctly specified. Furthermore, the AUC of this model was 0.77, which indicates a moderate discriminative capacity.

**Table 4:** Results of multivariate logistic regression model for overall study cohort.

Overall study cohort (n = 6,165)	OR (95%CI)	p-value
**Age ≥ 65**	1.80 (1.45–2.23)	0.000
**Presence of diabetes**	0.83 (0.65–0.99)	0.044
**Malignancy**	3.27 (2.58–4.15)	0.000
**Chemotherapy**	1.17 (0.86–1.61)	0.321
**Hemodialysis**	1.66 (1.17–2.35)	0.004
**Liver disease**	1.37 (1.06–1.77)	0.018
**Admission blood glucose ≥200mg/dL**	1.54 (1.19–1.99)	0.001
**Admission blood glucose ≤100mg/dL**	2.04 (1.55–2.68)	0.000
**Genitourinary infection**	0.59 (0.47–0.74)	0.000
**Skin infection**	0.72 (0.57–0.92)	0.008
**Abdominal infection**	0.64 (0.49–0.82)	0.001
**Other infections**	2.09 (1.57–2.78)	0.000

OR, adjusted odds ratio of mortality; CI, confidence interval; Goodness-of-fit test, p = 0.426.

**Table 5:** Results of multivariate logistic regression model for glucose variability subgroup.

Glucose variability sub-cohort (n = 1,537)	OR (95% CI)	p-value
**Age ≥ 65**	1.25 (0.77–2.02)	0.360
**Presence of diabetes**	0.57 (0.37–0.87)	0.009
**Malignancy**	2.29 (1.43–3.67)	0.001
**Chemotherapy**	1.44 (0.71–2.91)	0.316
**Hemodialysis**	1.17 (0.67–2.05)	0.573
**Liver disease**	1.21 (0.76–1.92)	0.417
**Sepsis-3**	3.85 (2.24–6.61)	0.000
**Admission blood glucose≥200mg/dL**	0.90 (0.59–1.36)	0.611
**Admission blood glucose ≤100mg/dL**	1.62 (0.93–2.84)	0.090
Glucose CoV<10%*	1.36 (0.76–2.43)	0.294
Glucose CoV>30%*	1.88 (1.24–2.86)	0.003
**Genitourinary infection**	0.58 (0.40–0.86)	0.007
**Skin infection**	0.64 (0.42–0.98)	0.041
**Abdominal infection**	0.51 (0.32–0.83)	0.007
**Other infections**	1.52 (0.80–2.89)	0.199

OR, adjusted odds ratio of mortality; CI, confidence interval; Sepsis-3, evidence of infection plus ΔSOFA (Sequential Organ Failure Assessment) score**≥**2; CoV, Coefficients of Variation, derived from standard deviation/mean of observations; Goodness-of-fit test, p = 0.156.

*Referece was Glucose CoV between 10% and 30%.

To examine how sepsis severity affected the outcomes of survival, the total study cohort was stratified into the sepsis-3 and non-sepsis-3 groups (S2 and S3 Tables). Elevated mortality risks were continuously seen in the elderly and the presence of malignancy in both groups. On the other hand, the presence of diabetes demonstrated significant reduction in aOR (0.63, 95% CI 0.42–0.95, p = 0.027) in the non-sepsis-3 cohort but had an insignificant impact on the sepsis-3 cohort (aOR = 0.90, 95% CI 0.70–1.16, p = 0.422), when sepsis became a dominated outcome driven factor.

## Discussion

The results of our study agreed with previous findings from western populations [10,18], and Asian cohorts by Tsai *et al*., (hazard ratio: 0.82) [19]; particularly, that diabetes is not an independent risk predictor for mortality for patients with sepsis. After controlling for age, underlying diseases, admission blood glucose level, and severity of sepsis, our diabetic patients with sepsis had a 22% to 47% less risk of in-hospital mortality than the non-diabetic group. By using sepsis-3 definition to further examine the influence of the presence of confirmed and progressing sepsis against clinically suspected sepsis, the conclusion continued to hold true (10% and 37% mortality risk reduction in diabetes). It is worth mentioning that our study cohort was characterized with a slightly higher ICU mortality rate, compared to the population-based study (27.3% vs. 22.6%),^6^ which can be explained by the nature of this tertiary referral center, where more complicated and severe patients are usually being referred and treated. In addition, the diabetic subgroup of this study should be regarded as a valid sample for nationwide diabetes representativeness, as the diagnosis of diabetes has been verified by linking the patients with their administrative claimed database, to check whether anti-diabetic medications were prescribed to treat their diabetes. The records shown that 77.4% of patients were prescribed with either oral anti-diabetic agents, insulin or combination therapy. This finding was aligned with the finding of Chang *et al*., [20], which found that that 60% of patients in the Taiwanese population with diabetes received anti-diabetic drugs treatment. This finding was through analysis of the 2009 national health insurance claimed database.

The lack of a strong influence of diabetes on the host response to sepsis-3 and survival was unexpected. Postulated mechanisms for this phenomenon include that patients with pre-existing diabetes may have adapted to chronic hyperglycemia induced oxidant stress over time [21]. Also, evidence suggests that diabetes may impair polymorphonuclear neutrophil cell function, alter cytokine regulation, and perhaps blunt inflammatory responses which slow down the fatal pathologic cascades of sepsis that progress from microcirculatory dysfunction, injury of vascular endothelium to massive secretion of cytokines and complement activation abruptly, resulting in reduce risk of mortality in acute sepsis [22].

Among our study patients, diabetic patients with increased levels of hyperglycemia upon ED admission possessed a similar mortality rate as the total patient cohort, and demonstrated a very slight difference in mortality (**≥**200 mg/dL: 7%; **≥**250 mg/dL: 7%, and **≥**300 mg/dL: 8%). Even though those with sepsis-3 had their odds ratio of death increase moderately (**≥**200 mg/dL: 10%; **≥**250 mg/dL: 11%, and **≥**300 mg/dL: 12%), no clear association between admission hyperglycemia and mortality was shown. On the contrary, non-diabetic patients had a significantly higher risk of mortality, both in patients with sepsis-3 (**≥**200 mg/dL: 18.1%; **≥**250 mg/dL: 20.3%, and **≥**300 mg/dL: 32.3%) and without sepsis-3 (**≥**200 mg/dL: 15%; **≥**250 mg/dL: 20%, and**≥**300 mg/dL: 28%). In emergency and critical care setting, the causes of acute hyperglycemia varied from patient to patient, sometimes it may simply because of hypertonic dehydration, parenteral or enteral nutrition feed, vasopressors or treatment induced elevated catecholamine levels after receive epinephrine for the relieve of acute asthma. Transient elevation of the blood glucose thus caused would have been normalized with correction of the underlying triggers. Dungan et al [23] categorized acute hyperglycemia into diabetic patients with deterioration of pre-admission daily glycemic control and hospital related hyperglycemia. The former is mainly caused either by β-cell dysfunction and/ or insulin resistance, while the latter is more related to stress hyperglycemia which is correlated with more severe disease complication, increased morbidity and mortality. The development of stress hyperglycemia involves a complex overlapping of counter-regulatory mechanism. The presence of large amount of lipopolysaccharides during sepsis activates the hypothalamus-pituitary-adrenal axis and the sympathoadrenal system, leading to increase output of cortisol and catecholamines, the circulating endotoxin stimulates the release of cytokines, these responses collectively exert multiple effects on the metabolic, cardiovascular and immune systems, primarily aimed at redistributing glucose from liver, muscle and fat to critical organs such as brain, central nervous system and blood cells of the immune system. During this instinct self-defense process, high hepatic glucose output via hepatic gluconeogenesis and glycogenolysis are believe to be the most important contributor to stress hyperglycemia. However, depends on the degree of hyperglycemia and duration of insults, the protective response may become harmful, the switch from insulin mediated glucose uptake to non-insulin mediated cellular glucose uptake increase oxidative stress, high glucose concentration may result in osmotic diuresis and volume depletion, especially in non-diabetes [24]. Therefore, sudden changes should be expected, early recognition and intervene may help preventing irreversible exacerbation.

On the other hand, initial hypoglycemia (admission glucose ≤100 mg/dL) significantly increases the risk of mortality, especially in those accompanied by sequentially deteriorating organ function, both in diabetic and non-diabetic septic patients. The causes of acute hypoglycemia episodes also vary from poor glycemic control, malnutrition, renal insufficiency, acute or chronic liver disease, and alcoholism, etc. Any of these causes may result in depleted glycogen storage and impaired gluconeogenesis, as mentioned above, the body’s self-defense mechanism requires a timely elevated glucose level to provide energy for organs' consumption to comeback the invasion effectively. A single episode of severe hypoglycemia may increase the risk of mortality by brain damage because of energy deficit during hypoglycemia. This may explain how both types of our patient fared worse in hypoglycemic episodes.

Glycemic control by insulin therapy has demonstrated beneficial effects on immune system and suppression of the release of inflammatory mediators, by avoiding the activation of the vascular endothelium and procoagulant state in preclinical findings [25]. Earlier studies suggested that non-diabetic patients with sustained hyperglycemia benefit greatly from tight glycemic control (target level of 80–110 mg/dL) by using insulin therapy, which can reduce mortality significantly among medical and surgical patients [26]. However, subsequent trial did not demonstrate the same benefit, as increased mortality was found in association with such tight control compared to a less strict target [27].

It is important to note that biochemical hypoglycemia occurred frequently among the patients in the medical ICU, and that tight glycemic control increased the incidence of hypoglycemia; which in turn, increased the vulnerability to severe hypoglycemia, and may offset some of the potential benefit of optimal blood glucose control. The NICE-Sugar study showed increased mortality (OR 1.14) for the group with strict glycemic control (target of 80 to 110 mg/dL) compared to the conventional group (target of <180 mg/dL) [26]. By adopting this conclusion, the Surviving Sepsis campaign [28] recommend the use of an upper target blood glucose≤ 180 mg/dL without a definite lower target except hypoglycemia. In principle, glycemic control should avoid blood glucose exceeding 180 mg/dL, but also pay attention to the development of hypoglycemia, and wide fluctuations in glucose levels which demonstrated an increased mortality, i.e., when a standardized approach for blood glucose management in ICU patients with severe sepsis is attempted, insulin administration can be considered when two consecutive blood glucose tests are exceed 180 mg/dL, also a less strict upper blood glucose target of ≤180 mg/dL rather than ≤ 110 mg/dL should be chosen. At the same time, bearing in mind that too rapid correction of longstanding pathological physical condition could be detrimental, which apply to both diabetes and non-diabetes patients.

As for how glucose variation impacted survival, the analysis derived from our subgroup demonstrated that wider glucose variation is a meaningful risk indicator. Patients with CoV higher than 30% or lower than 10% tended to have higher mortality rate, compared to patient with CoV between 10% & 30% (12% or 11% vs. 7%, respectively). Among the CoV>30% group, diabetes accounted for the majority of this subgroup, and a large portion (63.7%) of patients also had a simultaneously elevated admission blood glucose ≥200 mg/dL; implying that besides unstable glucose level during the hospitalization period, the daily blood glucose of these patients had not been properly controlled already. This probably contributed to the poorer prognosis. At the same time, non-diabetic patients having CoV>30% and elevated admission blood glucose≥ 200 mg/dL possessed an even higher risk of death. Probably, a more advanced disease status than the diabetes had been developed under the same range of glycemic level; the hypoperfusion and dysfunction of hemodynamic stabilized organs (liver and kidney) led to an impaired responsiveness of counter-regulatory hormones, making stable and normoglycemia difficult to achieve. This is in line with the published results which shown that hyperglycemia and glucose variability seem to exert less adverse influence on diabetic patients compared to non-diabetic patients [29–31]. Concerning patients with CoV<10%, although stable blood glucose with very little fluctuation should not be considered a warning signal during the hospitalization period, about one-third of our low CoV patients had admission glucose ≥ 200 mg/dL, indicating their blood glucose failed to be controlled and remained elevated throughout the hospitalization period.

It should be note that selection bias may exist in the glucose variability subgroup. As most of the subgroup of 1,537 patients with at least two blood glucose tests performed during the 48 hour emergency admission were diabetes (83.3%), diabetic patients usually undergo more frequent blood glucose monitoring by physicians, especially during ED stay, while non-diabetic patients usually receive only one baseline blood glucose check at ER admission, those receiving more frequent blood glucose monitoring may represent a group with more complicated underlying diseases or critically ill conditions other than diabetes.

Surviving Sepsis Campaign resuscitation and management bundles were introduced in our institute after a national education program conducted by the Joint Taiwan Critical Care Medicine Committee since 2004. The mortality rate of our study cohort is comparable to Levy *et al*. [32], who reported a slowly decreasing mortality observed after the resuscitation bundles were adopted and implement with good compliance. We believe that beside protocol implementation [33], continuous education and compliance review [34] has been shown to effectively change clinician behaviors and resulted in a reduction of severe sepsis mortality rate. Further research to validate the associations between glucose abnormality and variability control and clinical outcomes, by applying variability analyses as support algorithms in practical clinical decision, can realize the role of this modifiable parameter to improve sepsis care.

Some limitations of this study must be considered. As the study was conducted retrospectively, the stratification of patients into diabetes and non-diabetes rendered two study subgroups that were not totally balance concerning the distribution of comorbidities. Also, the accuracy of sepsis diagnoses was subject to uncertainties, since the sepsis patients of this study were enrolled based on a pragmatic definition of two sets of blood culture and chart review to confirm the existence of a focus of infection, patients with local infections may have been accidentally enrolled and introduced bias to the study outcomes. Nevertheless, using sepsis-3 definition to re-define sepsis subgroups has helped eliminating selection bias and improving the robustness of the results. There may still be a small portion of undiagnosed diabetes being grouped into the non-diabetic group. Although we have validated the diagnosis for each study patient by checking the chief complaints in the medical chart besides the ICD codings, HbA1c data would have been valuable to discriminate between stress hyperglycemia and undiagnosed diabetes in the non-diabetes group; unfortunately, this data is not available for non-diabetic patients. Even with these adjustments, it is possible that unmeasured confounders exist but cannot be identified or controlled by selection. Despite this limitation, our data reinforce the current existing knowledge and add updated information to the context of what was already known about how blood glucose and its variability influence survival outcome.

## Conclusions

Our study data, collected from over six thousand treated patients, indicated that while preexisting diabetes mellitus did not seems to exert negative impact both in clinically suspected sepsis and sepsis-3 patients, hyper- or hypoglycemia on ED admission and increase blood glucose variation during hospital stays were independently associated with higher odds ratio of mortality, especially among the non-diabetic sepsis patient.

## Supporting Information

S1 Table Patient characteristics of the glucose variability subgroup. (DOCX)

S2 Table Results of multivariate logistic regression model for the sepsis-3 subgroup. (DOCX)

S3 Table Results of multivariate logistic regression model for the non-sepsis-3 subgroup. (DOCX)
