# Supplementary material for: Association of In-Hospital Mortality and Dysglycemia in Septic Patients
Source: PLoS One. 2017 Jan 20;12(1):e0170408. doi: 10.1371/journal.pone.0170408 (PMC5249165; doi:10.1371/journal.pone.0170408)
Supplement: S1 Table — (DOCX) [file pone.0170408.s001.docx]

**S1 Table.** **Patient characteristics of the glucose variability subgroup**

|  | **All patient (n=1,537)** | | **Non-survivor (n=146)** | | **Survivor (n=1,391)** | |  |  |  | **Diabetes (n=1,281)** | | **Non-diabetes (n=256)** | |  |  |  |
| --- | --- | --- | --- | --- | --- | --- | --- | --- | --- | --- | --- | --- | --- | --- | --- | --- |
|  | Median or N | (IQR) or % | Median or N | (IQR) or % | Median or N | (IQR) or % | p-value | Odds* | 95%CI | Median or N | (IQR) or % | Median or N | (IQR) or % | p-value | Odds** | 95%CI |
| **Demographic & Comorbidities** | | | | | | | | | | | | | | | | |
| **Age, yr.** |  | (59-80) | 75.5 | (64-81) | 70 | (59-79) | <0.01 | 1.02 | (1.01-1.03) | 71 | (60-79) | 72 | (56.5-81.5) | <0.05 | 0.719 | (0.99-1.01) |
| **>65 years** | 957 | 62.3 | 103 | 70.5 | 854 | 61.4 | <0.05 | 1.51 | (1.04-2.18) | 802 | 62.6 | 155 | 60.5 | <0.05 | 0.535 | (0.83-1.44) |
| **Diabetes** | 1,281 | 83.3 | 101 | 69.2 | 1,180 | 84.8 | <0.001 | 0.40 | (0.27-0.59) | - | - | - | - | - | - | - |
| **Malignancy** | 268 | 17.4 | 49 | 33.6 | 219 | 15.7 | <0.001 | 2.70 | (1.86-3.92) | 211 | 16.5 | 57 | 22.3 | <0.05 | 0.690 | (0.50-0.96) |
| **Chemotherapy** | 96 | 6.2 | 18 | 12.3 | 78 | 5.6 | <0.01 | 2.37 | (1.37-4.08) | 73 | 5.7 | 23 | 9 | <0.05 | 0.610 | (0.38-1.00) |
| **Liver disease** | 266 | 17.3 | 35 | 24 | 231 | 16.6 | <0.05 | 1.58 | (1.06-2.37) | 204 | 15.9 | 62 | 24.2 | <0.001 | 0.590 | (0.43-0.82) |
| **Laboratory** | | | | | | | | | | | | | | | | |
| **RBC, 10^3^/uL** | 3.95 | (3.4-4.45) | 3.445 | (3.01-4.01) | 3.99 | (3.48-4.48) | <0.001 | 0.49 | (0.40-0.61) | 3.96 | (3.44-4.46) | 3.87 | (3.33-4.48) | <0.05 | 1.080 | (0.92-1.28) |
| **Hemoglobin, g/dL** | 11.4 | (9.8-13) | 10.3 | (8.9-11.6) | 11.6 | (9.9-13.2) | <0.001 | 0.8 | (0.74-0.87) | 11.4 | (9.8-13) | 11.5 | (10-13.1) | <0.05 | 0.990 | (0.94-1.05) |
| **MCHC, g/dL** | 33.3 | (32.3-34.2) | 33.1 | (31.7-34.1) | 33.3 | (32.4-34.2) | <0.05 | 0.9 | (0.80-1.01) | 33.3 | (32.4-34.2) | 33.3 | (32.2-34.1) | <0.05 | 1.050 | (0.96-1.15) |
| **RDW** | 14 | (13.1-15.5) | 15.8 | (14.2-17.6) | 13.9 | (13-15.2) | <0.001 | 1.29 | (1.22-1.37) | 14 | (13.0-15.4) | 14.4 | (13.3-16.5) | <0.001 | 0.880 | (0.83-0.92) |
| **Eosinophils,%** | 0.1 | (0-1) | 0 | (0-0.7) | 0.1 | (0-1) | <0.001 | 0.82 | (0.70-0.97) | 0.1 | (0-1) | 0 | (0-0.9) | <0.01 | 1.130 | (1.01-1.25) |
| **Bandemia,%** | 2.4 | (1-6) | 4 | (1.5-10) | 2 | (1-5) | <0.01 | 1.06 | (1.03-1.09) | 2 | (1-5) | 4 | (2-9) | <0.01 | 0.950 | (0.92-0.98) |
| **C-reactive protein** | 71.59 | (22.9-172.1) | 120.88 | (47.3-201) | 68.41 | (22.-169.8) | <0.01 | 1.002 | (1.00-1.01) | 69.5 | (21.7-164.4) | 111.5 | (29.8-198) | <0.01 | 0.998 | (0.996-0.99) |
| **Lactate, mg/dL** | 27.9 | (16.4-54) | 46.4 | (24.4-85.2) | 25.4 | (15.3-40.4) | <0.001 | 1.01 | (1.01-1.02) | 27.9 | (16.4-46.4) | 29.3 | (16.5-64.2) | <0.05 | 0.996 | (0.99-1.00) |
| **Admission blood glucose, mg/dL** | 203 | (141-304) | 179.5 | (112-299) | 206 | (147-305) | <0.01 | 0.999 | (0.99-1.00) | 211 | (153-319) | 159 | (118-222) | <0.001 | 1.004 | (1.00-1.01) |
| **BUN,mg/dL** | 25.7 | (15.7-44.9) | 45.1 | (23.6-68.1) | 23.4 | (14.9-41.2) | <0.001 | 1.02 | (1.01-1.02) | 25.7 | (15.7-44.6) | 25.7 | (14.6-46) | <0.05 | 0.997 | (0.99-1.00) |
| **Sodium, mEq/L** | 136 | (132-139) | 134 | (130-138) | 136 | (132-139) | <0.05 | 0.97 | (0.94-1.01) | 136 | (132-139) | 135 | (131-139) | <0.05 | 1.010 | (0.99-1.04) |
| **Albumin, g/dL** | 3.03 | (2.56-3.50) | 2.655 | (2.26-3.08) | 3.14 | (2.7-3.58) | <0.001 | 0.37 | (0.22-0.63) | 3.03 | (2.6-3.5) | 3.03 | (2.56-3.49) | <0.05 | 0.970 | (0.61-1.54) |
| **Total bilirubin, mg/dL** | 1 | (0.6-1.8) | 1.9 | (0.9-4.9) | 0.9 | (0.6-1.6) | <0.001 | 1.19 | (1.10-1.28) | 0.9 | (0.6-1.6) | 1.3 | (0.7-3.3) | <0.001 | 0.900 | (0.85-0.96) |
| **Prothrombin time, mL** | 13.3 | (12.1-15.8) | 16 | (13.9-23.8) | 12.9 | (11.9-14.6) | <0.001 | 1.07 | (1.04-1.11) | 13.2 | (12.0-15.3) | 13.8 | (12.2-18.7) | <0.05 | 0.980 | (0.96-1.00) |
| **INR, sec** | 1.2 | (1.1-1.4) | 1.5 | (1.3-2.1) | 1.2 | (1.1-1.3) | <0.001 | 1.92 | (1.42-2.60) | 1.2 | (1.1-1.4) | 1.3 | (1.1-1.7) | <0.05 | 0.850 | (0.66-1.08) |
| **AST,IU/L** | 37 | (24-72) | 65 | (37.5-142.5) | 34 | (23-63) | <0.001 | 1.001 | (1.00-1.01) | 34 | (23-62.5) | 60 | (37-142) | <0.001 | 0.999 | (0.99-1.00) |
| **Sepsis severity** | | | | | | | | | | | | | | | | |
| **Fever** | 1,428 | 92.9 | 126 | 86.3 | 1,302 | 93.6 | <0.01 | 0.43 | (0.26-0.72) | 1,200 | 93.7 | 228 | 89.1 | <0.01 | 1.820 | (1.16-2.86) |
| **Chill** | 486 | 31.6 | 19 | 13 | 467 | 33.6 | <0.001 | 0.3 | (0.18-0.49) | 435 | 34 | 51 | 19.9 | <0.001 | 2.070 | (1.49-2.87) |
| **Bacteremia** | 403 | 26.2 | 51 | 34.9 | 352 | 25.3 | <0.05 | 1.58 | (1.10-2.27) | 317 | 24.7 | 86 | 33.6 | <0.01 | 0.650 | (0.49-0.87) |
| **ICU admission** | 157 | 10.2 | 48 | 32.9 | 109 | 7.8 | <0.001 | 5.76 | (3.87-8.57) | 111 | 8.7 | 46 | 18 | <0.001 | 0.430 | (0.30-0.63) |
| **Sepsis-3** | 918 | 59.7 | 127 | 87 | 791 | 56.9 | <0.001 | 5.07 | (3.09-8.31) | 733 | 57.2 | 185 | 72.3 | <0.001 | 0.510 | (0.38-0.69) |
| **Steroid usage in ER** | 158 | 10.3 | 17 | 11.6 | 141 | 10.1 | <0.05 | 1.17 | (0.68-1.99) | 121 | 9.4 | 37 | 14.5 | <0.05 | 0.620 | (0.42-0.92) |
| **Steroid usage in OPD** | 12 | 0.8 | 0 | 0 | 12 | 0.9 | <0.05 | - | - | 10 | 0.8 | 2 | 0.8 | <0.05 | 0.990 | (0.40-0.78) |
| *IQR, interquartile range; RBC, red blood cell; MCHC, mean corpuscular hemoglobin concentration ; RDW, red blood cell distribution; BUN, blood urine nitrogen; INR, international normalized ratio; ICU, intensive care unit; *Non- survivor vs. survivor ;** Diabetes vs. non-diabetes* | | | | | | | | | | | | | | | | |
