# Supplementary material for: Association of In-Hospital Mortality and Dysglycemia in Septic Patients
Source: PLoS One. 2017 Jan 20;12(1):e0170408. doi: 10.1371/journal.pone.0170408 (PMC5249165; doi:10.1371/journal.pone.0170408)
Supplement: S2 Table — (DOCX) [file pone.0170408.s002.docx]

**S2 Table. Multivariate logistic regression model to evaluate associations between demographic, comorbidities, and dysglycemia with mortality in the sepsis-3 subgroup**

| **Sepsis-3 patient (n=2853)** | **OR (95% CI)** | **p-value** |
| --- | --- | --- |
| **Age>65** | 1.32 (1.02-1.71) | 0.034 |
| **Presence of diabetes** | 0.90 (0.70-1.16) | 0.422 |
| **Malignancy** | 2.42 (1.82-3.22) | 0.000 |
| **Chemotherapy** | 1.33 (0.89-2.00) | 0.162 |
| **Hemodialysis** | 1.16 (0.81-1.65) | 0.424 |
| **Liver disease** | 1.07 (0.80-1.42) | 0.659 |
| **Admission glucose≥200 mg/dL** | 1.30 (0.97-1.74) | 0.083 |
| **Admission glucose≤100 mg/dL** | 2.19 (1.59-3.03) | 0.000 |
| *OR, adjusted odds ratio; CI, confidence interval.* | | |
