# Supplementary material for: Association of In-Hospital Mortality and Dysglycemia in Septic Patients
Source: PLoS One. 2017 Jan 20;12(1):e0170408. doi: 10.1371/journal.pone.0170408 (PMC5249165; doi:10.1371/journal.pone.0170408)
Supplement: S3 Table — (DOCX) [file pone.0170408.s003.docx]

**S3 Table. Results of multivariate logistic regression model for the non-sepsis-3 subgroup**

| **Non-sepsis-3 patients (n=3,312)** | **OR (95% CI)** | **p-value** |
| --- | --- | --- |
| **Age>65** | 1.59 (1.07-2.36) | 0.022 |
| **Presence of diabetes** | 0.63 (0.42-0.95) | 0.027 |
| **Malignancy** | 8.19 (5.18-12.93) | 0.000 |
| **Chemotherapy** | 1.14 (0.69-1.86) | 0.615 |
| **Hemodialysis** | 0.43 (0.06-3.21) | 0.408 |
| **Liver disease** | 1.05 (0.57-1.94) | 0.87 |
| **Admission glucose ≥200 mg/dL** | 1.53 (0.90-2.60) | 0.115 |
| **Admission glucose ≤100 mg/dL** | 1.47 (0.84-2.57) | 0.178 |
| *OR, adjusted odds ratio of mortality; CI, confidence interval.* | | |
